# Attenuating Spinal Cord Injury by Conditioned Medium from Bone Marrow Mesenchymal Stem Cells

**DOI:** 10.3390/jcm8010023

**Published:** 2018-12-25

**Authors:** May-Jywan Tsai, Dann-Ying Liou, Yan-Ru Lin, Ching-Feng Weng, Ming-Chao Huang, Wen-Cheng Huang, Fan-Wei Tseng, Henrich Cheng

**Affiliations:** 1 Neural Regeneration Laboratory, Department of Neurosurgery, Neurological Institute, Taipei Veterans General Hospital, Taipei 11217, Taiwan; mjtsai2@vghtpe.gov.tw (M.-J.T.); dragonspruce@gmail.com (D.-Y.L.); yanlumiku@gmail.com (Y.-R.L.); mchuang@vghtpe.gov.tw (M.-C.H); wchuang@vghtpe.gov.tw (W.-C.H.); koli@livemail.tw (F.-W.T.); 2 Department of Life Science and Institute of Biotechnology, National Dong Hwa University, Hualien 97401, Taiwan; cfweng@gms.ndhu.edu.tw; 3 Department of Medicine, National Yang-Ming University, Taipei 11217, Taiwan; 4 Institute of Pharmacology, National Yang-Ming University, Taipei 11217, Taiwan

**Keywords:** stem cell, released factors, spinal cord injury, neuroprotection

## Abstract

Spinal cord injury (SCI) is a devastating neurological condition and might even result in death. However, current treatments are not sufficient to repair such damage. Bone marrow mesenchymal stem cells (BM-MSC) are ideal transplantable cells which have been shown to modulate the injury cascade of SCI mostly through paracrine effects. The present study investigates whether systemic administration of conditioned medium from MSCs (MSCcm) has the potential to be efficacious as an alternative to cell-based therapy for SCI. In neuron-glial cultures, MSC coculture effectively promoted neuronal connection and reduced oxygen glucose deprivation-induced cell damage. The protection was elicited even if neuron-glial culture was used to expose MSCcm, suggesting the effects possibly from released fractions of MSC. In vivo, intravenous administration of MSCcm to SCI rats significantly improved behavioral recovery from spinal cord injury, and there were increased densities of axons in the lesion site of MSCcm-treated rats compared to SCI rats. At early days postinjury, MSCcm treatment upregulated the protein levels of Olig 2 and HSP70 and also increased autophage-related proteins in the injured spinal cords. Together, these findings suggest that MSCcm treatment promotes spinal cord repair and functional recovery, possibly via activation of autophagy and enhancement of survival-related proteins.

## 1. Introduction

Spinal cord injury (SCI) is one of the most debilitating traumas affecting humans and might even result in death [1]. Severe SCI often permanently damages the spinal cord and results in the loss of sensory and motor function below the lesion site. Injury to the spinal cord results in immediate (primary) injury followed by a secondary phase of tissue damage [2,3]. Primary damage in the spinal cord is associated with neural destruction and vascular structure injury [4]. The secondary injury takes place during a period of a few minutes to several days after the primary injury, and involves the release of cytotoxic factors, edema, decreased blood flow, and accelerated apoptosis [5]. Because limited regenerative capability and the presence of growth inhibitors in the injury milieu, effective treatments for SCI are not currently available.

While there are no effective pharmacological interventions, cell-based therapy has become an appealing alternative. Bone marrow mesenchymal stem cells (BM-MSCs) are the most extensively used stem cells in tissue engineering due to their easy access, rapid ex vivo expansion, and poor immunogenicity [6,7]. Increasing evidence has shown that the transplanted MSC significantly promotes functional recovery after central nervous system damage in several animal models [8,9]. Spinal cord injury appears to be amenable to stem cell therapy. BM-MSCs could improve motor function recovery and increase angiogenesis in the spinal cord after SCI in rats [10]. BM-MSCs might create a favorable environment for regeneration, and express beneficial bioactive factors. However, BM-MSC survival and differentiation within the host tissues remains poor. It has been reported that less than 1% of MSCs survive for more than one week after systemic administration [11,12,13,14]. This suggests that the main effects of MSCs are probably mediated by paracrine mechanisms [15,16]. MSCs have a strong capacity for secretion of extracellular vesicles called exosomes which are suspected to participate in paracrine cellular communication [17]. MSC exosomes were applied and have been reported to mediate the therapeutic efficacy of MSC against several diseases or in models for traumatic brain injury, stroke and spinal cord injury [18,19,20,21,22]. 

Conditioned media from MSCs promoted neuronal survival through activation of the phosphoinositide 3-kinase/Akt pathway when administered even after injury [23]. Beneficial treatment of SCI rats with intrathecal (IT) administration of BM-MSC condition medium (MSCcm) has recently been reported [24,25]. Intrathecal delivery can be undertaken by pre-implanted IT catheter for repeated infusions or repeated injections by lumbar puncture; both are invasive. To be more clinically amenable use of MSCcm, this study was undertaken to investigate whether systemic administration of MSC-conditioned medium affects axonal connection and functional restoration after SCI. In vitro culture experiments were conducted in parallel to assess the beneficial properties of MSCcm. We propose that repeated MSCcm intravenous treatments of spinal cord injured rats will result in significant improvements in functional outcomes in the rat SCI model.

## 2. Experimental Section

### 2.1. Reagents and Antibodies

Cultured medium, fetal bovine serum (FBS), serum-free supplements and antibiotics were purchased from Gibco (NY, USA). Tissue culture plastics were from BD Bioscience (San Jose, CA, USA). Millicell culture inserts were from Millipore (Watford, UK). Primary antibodies and suppliers were: rabbit or mouse anti-neuronal class III beta tubulin (Covance, NJ, USA), rabbit anti- glial fibrillary acidic protein (GFAP) (Dako Cytomation, Ely, UK), mouse anti-HSP70 and goat anti-beta-actin (Santa Cruz Biotechnology, Dallas, TX, USA), mouse anti-MAP 2 and rabbit anti- Olig 2 (Millipore, Darmstadt, Germany), rabbit anti-LC3A (Novus Biologicals, Centennial, CO, USA), rabbit anti-p62/SQSTM1 (Proteintech Group, Chicago, IL, USA), and rabbit anti-Thioredoxin peroxidase II (AbFrontier, Seoul, Korea). Lactate dehydrogenase (LDH) kit was from Promega (Madison, WI, USA). Unless stated otherwise, all other chemicals were purchased from Sigma-Aldrich Co (St. Louis, MO, USA).

### 2.2. Preparation of Mesenchymal Stem Cells from Bone Marrow (BM-MSC) and Its Derivatives

BM-MSCs were prepared according to our previously described methods with modifications [26,27]. Bone marrow cells were flushed out from femur bone of adult Sprague Dawley (SD) rats with phosphate buffered saline (PBS; Gibco, NY, USA) and filtered through nylon cloths (70 μm sieve). The filtered cells were pelleted at 326× *g* for 10 min and resuspended with Dulbecco’s modified Eagle’s medium containing F12 (DMEM/F12; Gibco). The resulting cell suspension was layered onto Ficoll-Paque solution (1.077 g/mL) and spun to deplete the residues of red blood cells, platelets, and plasma. Ficoll-fractionated mononuclear cells were recovered from the gradient interface. The isolated cells were seeded in 75 cm^2^ flask (Falcon) and maintained in DMEM/F12 supplemented with 10% fetal calf serum (FCS), 100 U/mL penicillin and 100 μg/mL streptomycin at 37 °C in a water saturated atmosphere of 5% CO_2_/95% air. Non-adherent cells were removed at 2 days after initial seeding. Cultures developed colonies of fibroblast-like cells within 2 weeks. The attached cells at about 80% confluence were subcultured and expanded. BM-MSC from 3 to 5 passages were processed for collection of BM-MSC conditioned medium (MSCcm). In brief, BM-MSC cultures were washed trice with PBS, refilled with DMEM/F12 (serum-free medium) and further incubated for 48 h. The conditioned medium was then collected and concentrated with a centrifugal filter device (5 kDa cut-off, Amicon Ultra, Millipore). The resulted MSCcm (~40-fold concentrated) were preserved in aliquots at −80 °C until use.

### 2.3. Characterization of BM-MSC and MSCcm

BM-MSC was characterized by its round-shape colonies of fibroblastic-like cells and expression of MSC markers, such as integrin (CD29), Thy-1 CD90 and CD44. Cell surface antigen phenotyping was performed on isolated and expanded bone marrow cells. We also analyzed a panel of specified rat proteins in MSCcm, aiming to reveal a view of molecules present in the released fraction of MSC. The relative expression levels of 34 soluble mouse proteins were determined in CM using cytokine antibody array (Abcam, Cambridge, UK) according to the manufacturer’s instructions. Antibody arrays were performed on 3 distinct MSCcm samples. Background staining and spot size were analyzed as recommended by the manufacturer. Spot pixel densities on developed X-ray film was collected and determined using a scanner and image J analysis software.

### 2.4. Neuronal/Glial Culture and Treatment

Neuron-glial cultures were prepared from cerebrocortical or spinal cord regions of embryonic Sprague-Dawley (SD) rat fetus at gestation days 14–17 as described in Tsai et al [28,29]. Briefly, fetal cortexes or spinal cords were dissociated with mixtures of papain/protease/deoxyribonuclease I (0.1%:0.1%:0.03%) and plated onto poly-lysine-coated multiwell plates or transwell inserts (Millipore, Watford, UK) and maintained in DMEM (Gibco, NY, USA) supplemented with N2 (Gibco, NY, USA, for serum-free condition) or with 10% FBS. The second day after cell seeding, treatment was applied to neuronal culture and incubated for 2–3 days. For oxygen glucose deprivation (OGD) induction, cultures were washed trice with PBS and maintained in glucose-free and serum-free DMEM. The cultures were then placed into an air-tight chamber which is regulated by a ProOx 110 oxygen controller (BioSpherix, Redfield, NY, USA) to obtain O_2_ free condition. After 3 h of OGD treatment, reperfusion was simulated by replacing the exposure medium with normal growth medium. Neuron-glial cultures were treated with MSC coculture or MSCcm at 0 h after OGD reperfusion or normoxic treatment. Two days after OGD treatment, cultures were harvested for estimation of viability including measurement of LDH release in medium and betaIII tubulin immunoreactive (IR) density. Image-Pro plus software (NIH systems, Bethesda, MD, USA) was employed for quantitative analysis of tubulin-IR neurite density. A commercial kit (CellTiter 96 Aqueous; Promega Corporation, Madison, WI, USA) was used for determining the extent of cell survival as cytosolic LDH release in cultured medium.

### 2.5. Neurite Outgrowth Assay

We performed analysis of axonal outgrowth at 3 days after culture treatment. Two methods were employed to measure the neurite-promoting effect of MSC or MSCcm. First, neuron-glial cultures were seeded on 1μm PET hanging transwell inserts (Millipore, Watford, UK) inside 24-well plate at a density 3 × 10^5^ cells/cm^2^, and maintained in growth medium with or without treatment. Only axons passed through the porous membrane and run parallel to cell body layer. After treatment, cells were fixed and processed for immuno-staining against betaIII tubulin. The cell bodies inside the transwell were wiped off, leaving the extending neurite intact on the other side of the transwell. Second, neuronal cultures were seeded to polylysine-coated plate at lower density 5 × 10^4^ cells/cm^2^, and treated with MSC or MSCcm. Beta III tubulin-IR neurite of cultured cells was analyzed at the end of treatment. The extending neurites was analyzed according to the method described in our previous article [30].

### 2.6. Contusive Spinal Cord Injury (SCI) and Treatment

SCI was induced in adult female SD rats using the New York University weight-drop device as described previously [29,31]. Animal handling and experimental protocols were carefully reviewed and approved by the animal studies subcommittee of Taipei Veterans Hospital (IACUC 2015-167 and IACUC 2016-291). Briefly, rats were anaesthetized and underwent a T8-10 laminectomy. A 10-g weight was allowed to drop 50 mm onto the exposed dura at the T9 vertebral level to produce a contusion injury. Control (sham) animals received only a laminectomy, exposing the cord without disturbing the dura. Following injury, the incision was closed and sutured. Within 30 min after contusive injury, the rats received first injection of 150 μL MSCcm (serum-free, 40-fold concentrated) through tail vein administration. Second and third vascular administrations of MSCcm to SCI rats were conducted at 2nd and 3rd day postinjury. To avoid urinary tract infection, manual emptying of the urinary bladder was carried out twice daily. Animals were subjected to evaluation of motor functions with the locomotor’s rating Basso, Beattie, Bresnahan (BBB) 21-point open field scale at certain time points and sacrificed for histology or biochemical studies.

### 2.7. Western Blot Analysis

After treatment, cultured cells were washed twice with PBS and solubilized in lysis buffer containing 40 mM Tris buffer (pH 7.5), 7 M urea/2M thiourea, 4% CHAPS, 1 mM PMSF, 1 mM Na_3_VO_4_, 1 mM dithiothreitol, and a protease inhibitor kit (BM, Mannheim, Germany), whereas spinal cord segments (T8-10) were homogenized in the same lysis buffer (500 μL) with the help of a sonicator. Protein was quantified by a Biorad protein assay reagent. Equal amounts of proteins were loaded and analyzed using 8–12% gels (SDS PAGE), as described previously [28,29].

### 2.8. Immunohistochemical Analysis

Frozen spinal sections were processed for immunohistochemistry following the method described in our recent articles [30,32]. The tissue sections or cultured cells were incubated with primary antibodies, followed by respective 2nd antibodies for histological evaluation as described. For double immunostaining, the secondary antibodies used were Alex 488 fluorophore donkey anti-rabbit antibody (Molecular Probes, Eugene, OR, USA) and Cy3-conjugated donkey anti-mouse antibody (Jackson ImmunoResearch Laboratories, West Grove, PA, USA). Primary antibody omission controls were performed for all immunostaining protocols to control for nonspecific binding. Fluorescent visualization and photography were performed on a Zeiss Axioscope microscope with appropriate filter sets (Zeiss, Oberkochen, Germany). 

## 3. Results

BM-MSCs used in the present study were prepared from adult rat bone marrow. The Ficoll-fractionated mononuclear cells were isolated, expanded and analyzed. Similar to the results shown in our previous article [27], more than 95% of isolated BM-MSC expressed β1-integrin (CD29) and Thy1 (CD90), typical MSC marker proteins, but failed to express CD34, a surface marker for early hematopoietic stem cells (Supplementary Figure S1). An antibody-based protein array analysis reacting to 32 rat cytokines was further used to investigate the protein components contained in MSCcm. Antibody arrays, performed on three distinct MSCcm samples, reveal the presence of >30 cytokines (Supplementary Figure S2). MSCcm composes of comparative higher levels of VEGF, TIMP-1, PDGFαα, LIX, MCP-1 and CINC-1 and -2α. These agreed with the published properties of MSCcm [24,26,27]. 

### 3.1. Effects of BM-MSC Cocultures on Neurite Outgrowth and OGD-Induced Neuronal Damage

**Figure 1:**
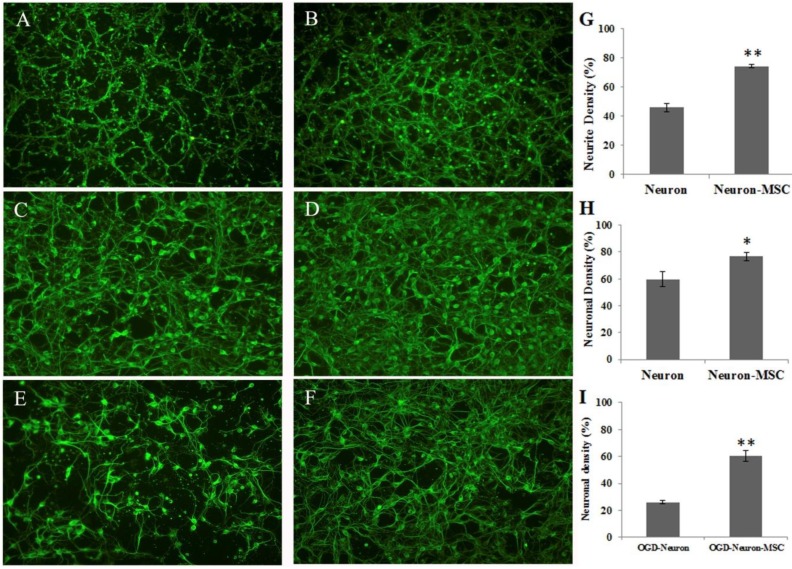
Beneficial effect of MSC co-cultures on neuronal connection and survivals. (**A**) Extended neurite of neuron–glial cultures through transwell. (**B**) Extended neurite of neuron–glial cultures through transwell in the presence of MSC cocultures. (**C**) Neurite outgrowth of neuron–glial cultures, grown on culture plate. (**D**) Neurite outgrowth of neuron–glial cultures in the presence of MSC-seeded transwell. (**E**) OGD-treated neuron–glial cultures, grown on culture plate. (**F**) OGD-treated neuron–glial cultures in the presence of MSC-seeded transwell. (**G**) Quantification of neurite outgrowth of A and B. (**H**) Quantification of neuronal density of C and D. (**I**) Quantification of neuronal density of E and F. Data represent the mean ± SEM. * *p* < 0.05, ** *p* < 0.01, indicates significant difference between neuron–glial cultures and cocultures of neuron–glia and MSC.

We first tested the beneficial effect of BM-MSC coculture with cortical neuron-glial cultures. Neuron-glial cultures were seeded to transwell inserts (1 μm porous membrane, Millicell) and cocultured with MSC. The tubulin-positive neurites that passed through the porous membrane were measured and quantified. Figure 1A–G shows that neuron–glial cultures readily extended neurites to the bottom of the insert. MSC coculture extensively induced neurite outgrowth in cortical neuron–glial cultures (*p* < 0.01). Alternatively, neuron–glial cultures were seeded to cultured plate and treated with OGD or normoxic conditions. The resulted cultures were then co-cultured with MSC that were grown in a transwell insert. Figure 1C,D,H demonstrates neurite outgrowth-improving effect of MSC cocultures (*p* < 0.05). Furthermore, MSC co-culture significantly protected neuron–glial cells from OGD-induced damage (*p* < 0.01), as shown in Figure 1E,F,I.

### 3.2. Effect of BM-MSC Conditioned Medium on Spinal Cord Neuron-glial Culture

**Figure 2:**
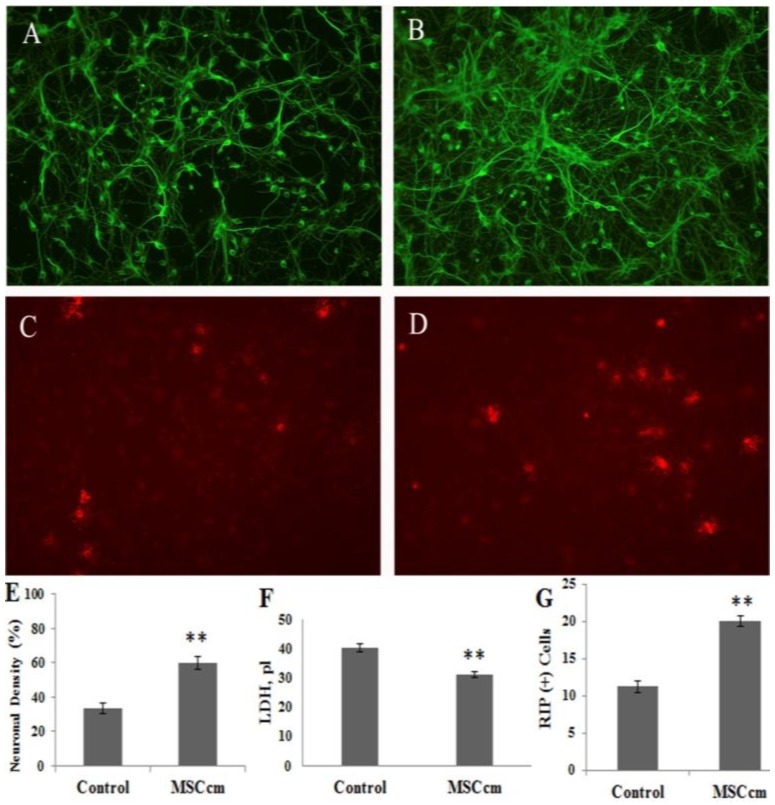
Beneficial effect of bone marrow mesenchymal stem cells (BM-MSC) conditioned medium (MSCcm) on cell survival in spinal cord neuron–glial cultures. (**A**) Beta III tubulin (+) neuronal connection of neuron–glial cultures. (**B**) Neuronal connection of MSCcm-treated neuron–glial cultures. (**C**) Rip (+) oligodendrocytes in neuron–glial cultures. (**D**) Oligodendrocytes in MSCcm-treated neuron–glial cultures. (**E**) Quantification of neuronal density of A and B. (**F**) Lactate dehydrogenase (LDH) release in the neuron–glial culture medium. (**G**) Quantification of oligodendroglial density of C and D. Data represent the mean ± SEM. ** *p* < 0.01, indicate significant difference between saline control and MSCcm treatment.

**Figure 3:**
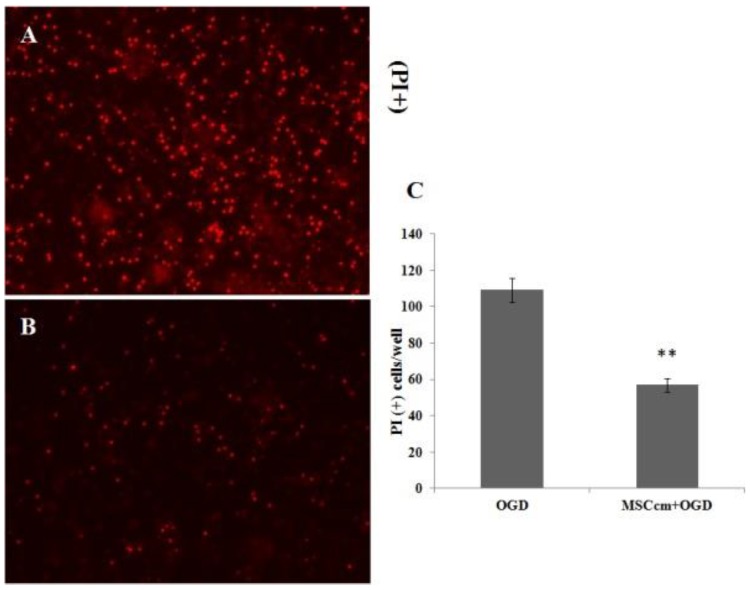
BM-MSC conditioned medium protected spinal cord neuron–glial cultures from OGD-induced damage. (**A**) OGD-induced neuron–glial cultures. (**B**) OGD-induced and MSCcm-treated neuron–glial cultures. (**C**) Quantification of propidium iodide (PI) positive cells of A and B. The results are reported as mean ± SEM. ** *p* < 0.01 indicates significant difference between saline + OGD and MSCcm + OGD treatment.

Figure 1 shows the beneficial effects of BM-MSCs which were not in direct contact with neuron–glial cultures, suggesting the effects were coming from the released fraction of MSC. We therefore prepared conditioned medium from BM-MSC (MSCcm) and further tested its beneficial effect on injured spinal cord neurons in cultures or in vivo. Addition of MSCcm to spinal cord neuron–glial cultures for three days significantly increased neuronal connection and oligodendroglial numbers but decreased LDH release to medium, indicating survival-enhancing effects of MSCcm (all *p* < 0.01; Figure 2). Furthermore, MSCcm markedly protected OGD-induced spinal cord neuron–glial cultures damage, as depicted by propidium iodide positive cells (Figure 3, *p* < 0.01).

### 3.3. Effect of BM-MSC Conditioned Medium in Contusive Spinal Cord Injured Rats

**Figure 4:**
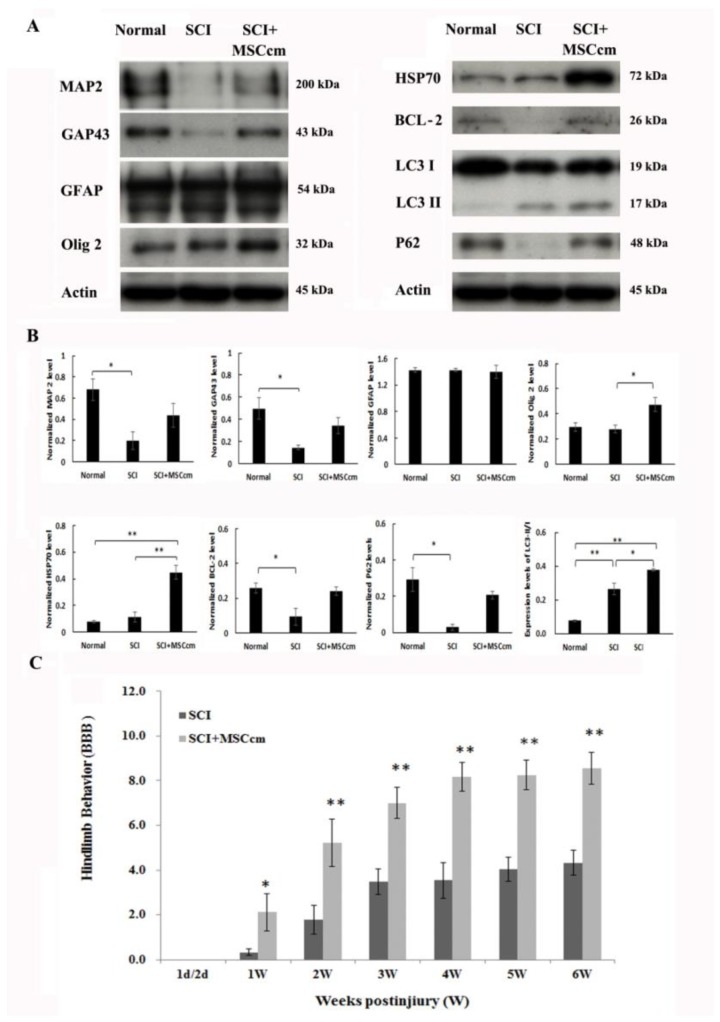
Expression of protein levels in spinal cord of rats and the hindlimb functional behaviors in SCI rats. (**A**) The protein expression levels, determined by Western blot analysis, in normal or injured epicenter of SCI rats at 7 days after the contusion injury. Representative blots are shown. (**B**) The bar graphs show the quantitative analysis of the protein expression relative to actin. Results were means ± SEM (*n* = 3 rats each). (**C**) Time course of hindlimb locomotor recovery in rats receiving saline or MSCcm after contusive injury. The time course changes of BBB in SCI rats that were administered saline or BM-MSCcm through tail vein injection. The hindlimb recovery of the animals was assessed in a double-blind manner. Locomotor recovery of SCI rats was evaluated over a 6-week period, using a 21-point scale (Basso, Beattie, Bresnahan (BBB) locomotor rating scale). The results are reported as mean ± SEM. Statistical significance was evaluated using one-way ANOVA and Bonferroni’s *t*-test. * *p* < 0.05, ** *p* < 0.01.

We next examined if the beneficial properties of MSCcm facilitated functional recovery in rats after spinal cord injury. Adult rats were subjected to contusive SCI. Within 30 min after injury, animals received first injection of either 150 μL saline or concentrated serum-free MSCcm via tail vein administration. Second and third MSCcm (or saline) vascular injections were conducted to rats at second and third day postinjury. At 7 days postinjury, a set of rat spinal cords were processed for Western blot analysis and the density of blots was quantified (Figure 4). Spinal cord injury significantly reduced protein expression of MAP 2, a neuronal marker. MSCcm treatment appeared to preserve the MAP 2 level to a certain degree (Figure 4A,B). Similarly, growth-associated protein 43 (GAP43) level was decreased by injury (*p* < 0.05) and has a tendency of preservation by MSCcm treatment. While astroglial GFAP levels were not altered among groups, oligo 2 levels were effectively increased in MSCcm-treated SCI rats, compared to that in SCI rats. Oligo 2 is a transcription factor playing essential roles in oligodendrocyte specification and differentiation [33]. Expression levels of antiapoptotic B-cell lymphoma 2 (BCL-2) were reduced by spinal cord injury (*p* < 0.05) but had a tendency of recovery by MSCcm treatment. Levels of heat shock protein (HSP) 70, a chaperone protein and also an exosomal marker, were significantly increased by MSCcm treatment, compared to normal or SCI rats (Figure 4, *p* < 0.01). In Supplementary Figure S3 the level of thioredoxin peroxidase (TPX) II was also increased by MSCcm treatment but did not reach significance. We also measured apoptosis- and autophagy-related proteins in the thoracic spinal cords. The level of caspase 3 (32 kDa) was significantly induced by SCI (*p* < 0.01) or by MSCcm-treated SCI (*p* < 0.05). However, there were no differences between cords of SCI and MSCcm-treated cords. To monitor activity of cell autophagy, microtubule-associated protein light chain (LC) II and p60 are most commonly used. Interestingly, levels of LC3 II (17 kDa), autophagosome localized protein, were induced by injury (*p* < 0.05) and further enhanced by MSCcm treatment after injury (*p* < 0.05), whereas LC3 I (19 kDa) levels were not altered among groups (Figure 4). Thus, the ratio of LC3-II/I was induced by injury (*p* < 0.01) and further increased by MSCcm treatment (*p* < 0.05) as shown in Figure 4A,B. Consistent with this result, levels of p62 (also known as Sequestosome 1 (SQSTM1)/sequestome1) was reduced by injury (*p* < 0.05). P62 levels trended higher in MSCcm-treated injured cords than in injured cord but did not reach significance (*p* = 0.064). 

SCI rats were weekly monitored for hindlimb behavior as an index for functional restoration. At 6 weeks postinjury, experimental rats were sacrificed for morphological analysis. Figure 4C shows that MSCcm-treated rats had better locomotion from first week postinjury onwards (*p* < 0.05). Furthermore, MSCcm-treated SCI rats maintained significantly higher levels of BBB scores, indicating functional recovery, throughout 6 weeks postinjury (2nd–6th weeks; all *p* < 0.01). 

**Figure 5:**
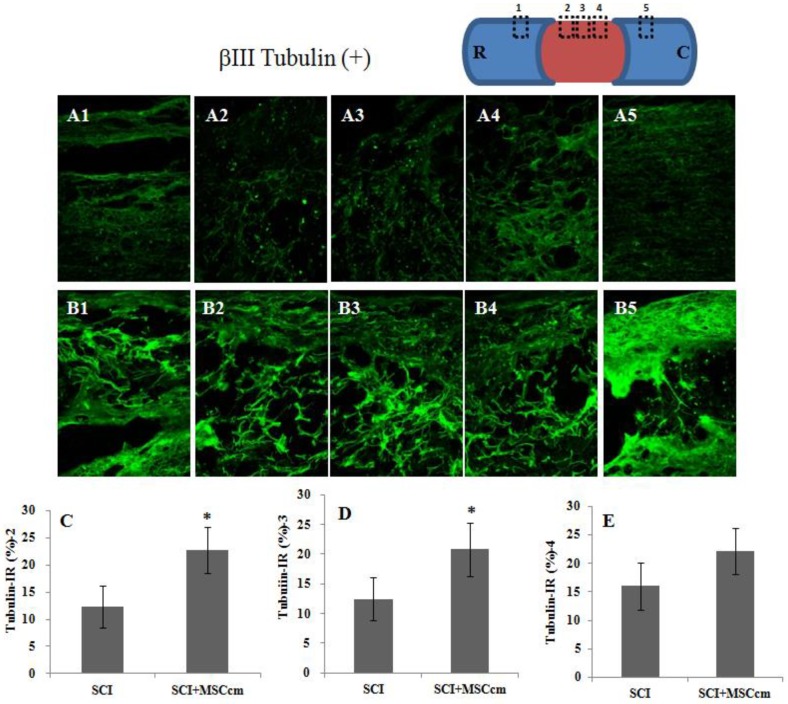
Tubulin-immunoreactive nerve fibers in the thoracic spinal cords of SCI rats at 6 weeks after injury. (**A**) Representative images of longitudinal spinal cord sections in SCI rat. (**B**) Representative images of spinal cord sections in MSCcm-treated SCI rat. (**C**–**E**) Quantification of tubulin-positive axons in area 1, 2 and 3, respectively, of spinal cord sections shown in A and B. Note the significant increase of tubulin positive nerve fibers in MSCcm-treated groups at 6 weeks postinjury. MSCcm was administered to SCI rats through tail vein injection after spinal cord contusion. After 6 weeks, immunofluorescence positive area of beta III tubulin was detected in a longitudinal section at the epicenter of the spinal cord. The results are reported as mean ± SEM. Statistical significance was evaluated using one-way ANOVA and Bonferroni’s *t*-test. * *p* < 0.05.

Histological assessment of injured spinal cord was done with immunofluorescent beta III tubulin staining in longitudinal spinal cord section taken from rats of SCI or SCI + MSCcm treatment (Figure 5A,B). Results showed that MSCcm-treated rats have higher axonal densities in the lesions at 6 weeks after SCI (Figure 5 (B2–B4) compared to Figure 5 (A2–A4)). Quantitative stereological analysis of tissue areas (2, 3, 4) reveals significant difference in areas 2 and 3 (*p* < 0.05) among samples studied (Figure 5C–E). MSCcm-treated rat spinal cord possessed more preserved tubulin (+) nerve fibers than those of SCI rats.

## 4. Discussion

Spinal cord injury is a severe disease and has a poor prognosis. The present study tests whether conditioned medium from MSC has similar beneficial effects on the recovery of injured spinal cord neurons as those of cell-based treatment. We present evidence supporting the notion that MSCcm reduced the extent of spinal cord neuronal injury both in vivo and in vitro. Cell culture study showed that the magnitude of the CM-induced beneficial effects was similar to that induced by MSC cocultures, highlighting the potential of a cell-free therapeutic approach for the treatment of injured spinal cord neurons in vivo.

We first confirmed the neurite promoting and neuroprotective effect of MSC co-cultures. Similar effect was observed even if MSCcm was added to spinal cord neuron–glial culture. Furthermore, rip (+) oligodendroglial numbers were significantly increased in MSCcm-treated cultures. This indicates that the beneficial effects of MSC were possibly derived from its released fraction. Cytokine array analysis on MSCcm demonstrated the presence of at least 30 cytokine/proteins (Supplementary Figure S2). The neurotrophic and protective functions of MSCcm are consistent with previous studies [24,25] and our report [27]. The in vivo study further demonstrated that MSCcm possessed therapeutic potential for the treatment of SCI. We used intravenous injection of MSCcm SCI rats to mimic the paracrine actions of BM-MSCs in vivo. Repeated administration of MSCcm to SCI rats significantly enhanced hindlimb behavior restoration. Supporting the observed neurological improvements, the nerve fibers (axons) were significantly preserved in the MSCcm-treated spinal cords as measured by immunohistochemical staining. The results suggested neuroprotective effects of MSCcm against spinal cord injury, and provided a rational basis for cell-free therapy. Importantly, MSCcm was able to preserve neuronal damage after contusive injury via systemic and vascular administration, not requiring intrathecal injection, of MSCcm. 

Changes of protein expression in normal or SCI rats were demonstrated by Western blot analysis conducted at 7 days postinjury. The density of blots was quantified and is shown in Figure 4. Axonal damage occurred following contusive injury, as evidenced by significant decreased MAP 2 levels in the injured cord (*p* < 0.05; Figure 4A). MAP 2 levels in MSCcm-treated cords trended higher but did not reach statistical significance. Similarly, GAP43 level was decreased by injury (*p* < 0.05) and has a tendency of preserved level in MSCcm-treated group. GAP43 is a protein expressed at high levels in neuronal growth cones during axonal regeneration. Expression levels of antiapoptotic B-cell lymphoma 2 (BCL-2) were reduced by spinal cord injury but had a tendency of recovery by MSCcm treatment. This demonstrates survival/regeneration-improving effects of MSCcm. Interestingly, levels of Olig 2 were effectively induced by MSCcm treatment. Oligo 2 is a transcription factor playing essential roles in oligodendrocyte specification and differentiation [33]. MSCcm might play some roles in oligodendrogliogenesis, as MSCcm treatment not only enhanced oligodendroglial survival in cultures but induced Olig 2 level in vivo. The underlying mechanism and effective molecules remain to be determined.

On the other hand, recent data suggest that the MSC-derived exosomes may deliver bio-active proteins to the injured cells [34]. The exosomes can pass the blood–brain barrier and play an important role in exchange of information between neurons and glial cells [35]. The MSCcm used in the present study was concentrated from the release fraction of MSC by 5 kDa cut-off centrifugation. Almost all bioactive factors and extracellular vesicles including exosomes in the MSCcm were thus retained and stored at −80 °C until use. It has been shown that exosomes are rich in heat-shock proteins (such as Hsp70 and Hsp90), mRNA and microRNA [36,37] and that HSP70 is released from cells in exosomes [38]. Intriguingly, increased HSP70 levels were found in MSCcm-treated cord but not in other groups, suggesting its possible origin of MSC-derived exosomes. Supporting this view is the increased TPXII levels in MSCcm-treated cords. The antioxidant protein TPXII, not having signal peptide, is secreted and associated with the exosomes [39,40,41]. This suggests that MSC-sourced exosomes are, at least in part, a mediator of the MSCcm associated therapeutic potencies. 

Autophagy is a cytosolic protective process of degrading misfolded proteins and damaged organelles under stress [42]. Autophagosome localized protein LC3 and p62 are commonly used to monitor activity of cell autophagy in SCI. Increased levels of p62 and LC3 II were found in the MSCcm-treated group, suggesting increased autophagy and a possible blockage of autophagosome clearance. p62 is incorporated into autophagosomes through binding to LC3 and subsequently degrades through autophagy [43]. LC3II expression is an index used to reflect the number of autophagic vacuoles [44]. The increased LCII/I levels in the injured cords are consistent with previous reports [45,46,47]. Many articles report that the ratio of LC3II/I is significantly increased in the lesion at 3 and 7 days after SCI [45,46,47]. Interestingly, systemic MSCcm administration further increased LC3 II/I levels in the spinal cord. The autophagy-promoting effects by MSC graft [48], MSCcm [49] or MSC exosomes [50,51] have been reported in culture or in vivo studies, although not have not been seen in an SCI rat model. Additional experiments are warranted to determine the relation and role of individual RNA, miRNA and related protein elements of exosomes and SCI autophagy.

MSCcm represents the complete regenerative milieu of cell-sourced secretome. The protective efficacy of intravenous MSCcm treatment in SCI rats would be multitudinous. Following intravenous administration, MSCcm bioactive substances are readily bioavailable and potentially affect both local and systemic physiological processes. The bioactive substances from MSCcm can modulate the immune and neuronal environments, triggering or repressing signaling events that modify the course of neuropathy. Enhancement of Olig 2 and HSP70 levels and activation of autophagy in the present study may contribute, at least in part, to the underlying beneficial effect of MSCcm. Taken together, in the present study, MSCcm treatment induced a long-lasting neuroprotective effect on SCI rats and may provide an environment more conducive to corticospinal axonal regrowth after spinal cord injury.

## Supplementary Materials

The following are available online at http://www.mdpi.com/2077-0383/8/1/23/s1, Figure S1: Characterization of rat bone marrow mesenchymal stem cells (BM-MSC), Figure S2: Factors revealed in conditioned medium of BM-MSC, Figure S3: Expression of protein levels in normal or injured spinal cord of rats at 7 days post-injury.
