# Supplementary material for: Attenuating Spinal Cord Injury by Conditioned Medium from Bone Marrow Mesenchymal Stem Cells
Source: J Clin Med. 2018 Dec 25;8(1):23. doi: 10.3390/jcm8010023 (PMC6352201; doi:10.3390/jcm8010023)
Supplement: Supplementary file 1 [file jcm-08-00023-s001.pdf]

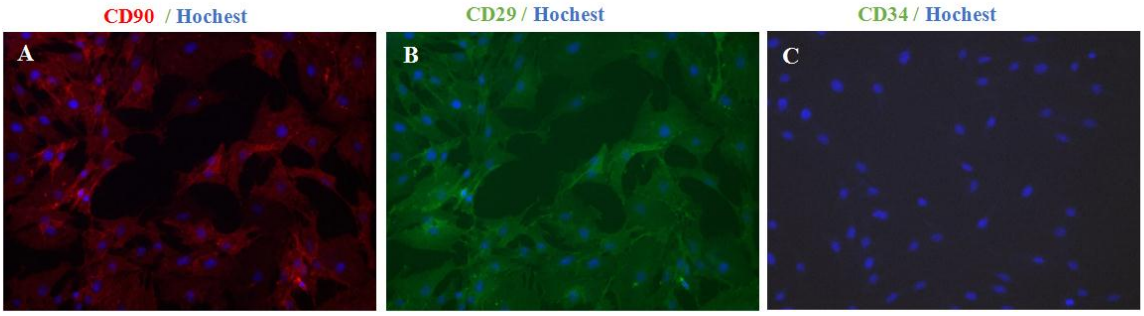


**Figure S1.** Characterization of rat bone marrow mesenchymal stem cells (BM-MSC). (**A**) CD90 immunoreactive cells with Hoechst. (**B**) CD29 immuno-reactive cells with Hoechst. (**C**) CD34 immunoreactive cells with Hoechst.

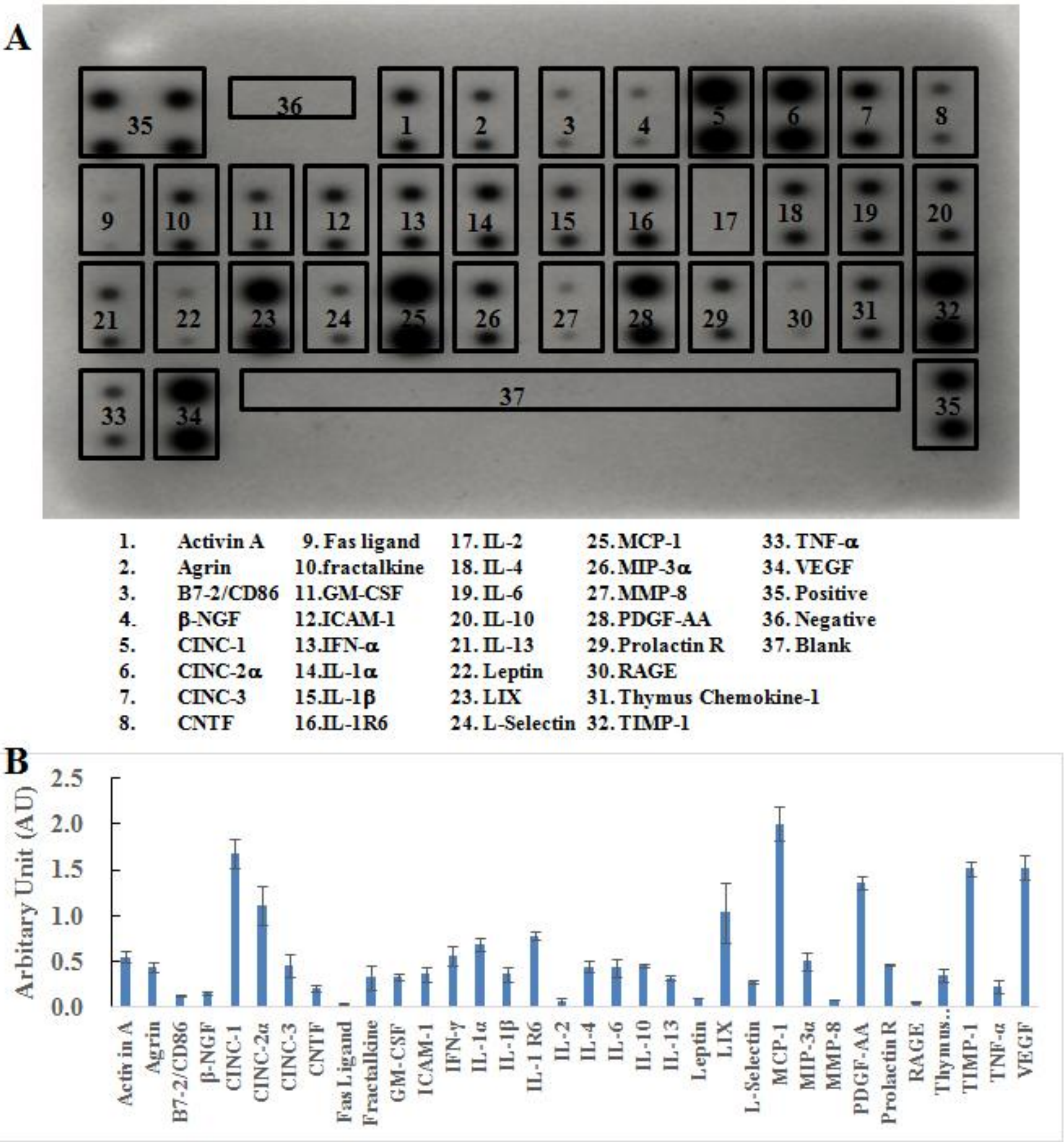


**Figure S2.** Factors revealed in conditioned medium of BM-MSC. (**A**) Cytokines array profile of 34 specified proteins of MSCcm. (**B**) Bar diagrams represents the ratio of the spot mean pixel densities/reference point pixel densities from 3 independent experiments. Antibody arrays were performed on 3 distinct CM samples. Results were means ± SEM.

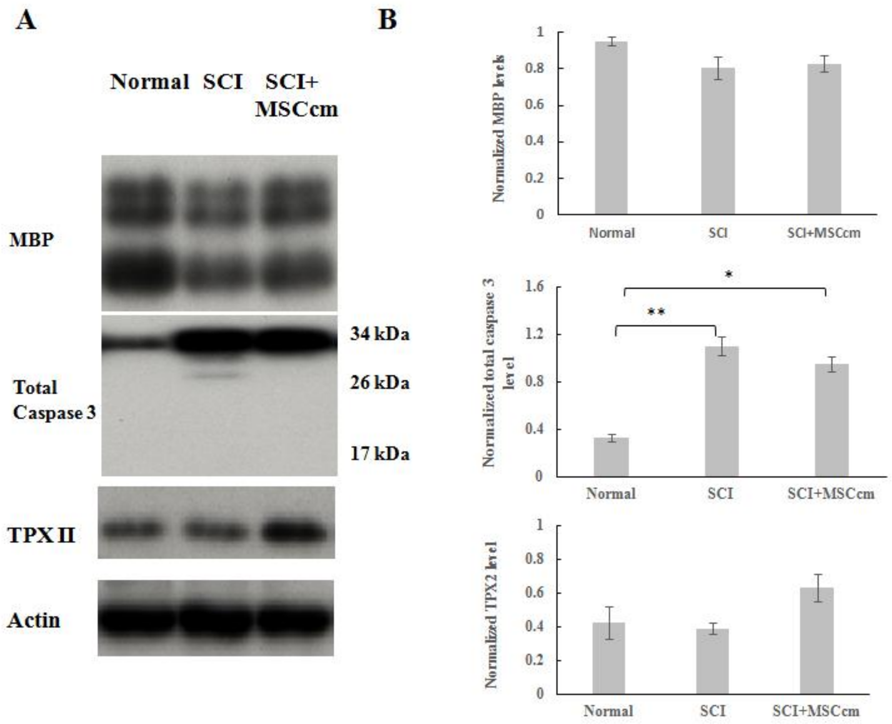


**Figure S3.** Expression of protein levels in normal or injured spinal cord of rats at 7 days post-injury. (**A**) Protein levels were determined in spinal cords by western blot analysis. (**B**) The density of blots was quantified and expressed as bar charts. Results were means ± SEM ($N$ =3 rats each). Statistical significance was evaluated using one-way ANOVA and Bonferroni's $t$-test. * $p < 0.05$,** $p < 0.01$.
